# 4-Cyclo­butyl­amino-3-nitro­benzoic acid

**DOI:** 10.1107/S1600536809021412

**Published:** 2009-06-13

**Authors:** Shivanagere Nagojappa Narendra Babu, Aisyah Saad Abdul Rahim, Hasnah Osman, Ching Kheng Quah, Hoong-Kun Fun

**Affiliations:** a School of Pharmaceutical Sciences, Universiti Sains Malaysia, 11800 USM, Penang, Malaysia; b School of Chemical Sciences, Universiti Sains Malaysia, 11800 USM, Penang, Malaysia; c X-ray Crystallography Unit, School of Physics, Universiti Sains Malaysia, 11800 USM, Penang, Malaysia

## Abstract

The asymmetric unit of the title compound, C_11_H_12_N_2_O_4_, contains two crystallographically independent mol­ecules with similar geometries. Both mol­ecules contain an intra­molecular N—H⋯O hydrogen bond. The dihedral angles between the benzene ring and the mean plane of the cyclo­butane ring are 38.29 (7) and 57.04 (8)° in the two mol­ecules, and the nitro group is twisted slightly away from the plane of the benzene ring [dihedral angles = 9.15 (12) and 9.55 (12)° in the two mol­ecules]. In the crystal, the independent mol­ecules are linked into dimers by O—H⋯O hydrogen bonds between their carboxyl groups, and C—H⋯O and C—H⋯π inter­actions are formed between dimers.

## Related literature

For the biological activity of benzimidazole derivatives, see: Wright (1951 ▶); Singh *et al.* (2009 ▶). For details of the synthesis, see: Narendra Babu *et al.* (2009*a* ▶,*b* ▶); Ishida *et al.* (2006 ▶).
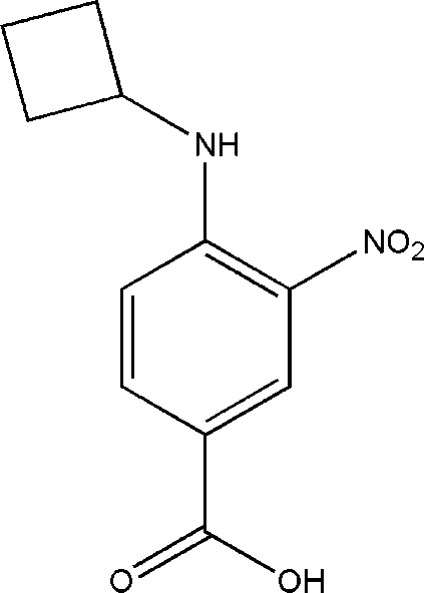


## Experimental

### 

#### Crystal data

C_11_H_12_N_2_O_4_
*M* *_r_* = 236.23
Triclinic, 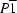

*a* = 9.8555 (2) Å
*b* = 10.5308 (2) Å
*c* = 10.9110 (2) Å
α = 74.860 (1)°
β = 78.265 (1)°
γ = 84.826 (1)°
*V* = 1069.44 (4) Å^3^
*Z* = 4
Mo *K*α radiation
μ = 0.11 mm^−1^
*T* = 110 K
0.37 × 0.23 × 0.21 mm

#### Data collection

Bruker SMART APEXII CCD diffractometer
Absorption correction: multi-scan (*SADABS*; Bruker, 2005 ▶) *T*_min_ = 0.960, *T*_max_ = 0.976
29344 measured reflections
7670 independent reflections
6094 reflections with *I* > 2σ(*I*)
*R*_int_ = 0.027

#### Refinement

*R*[*F* ^2^ > 2σ(*F* ^2^)] = 0.048
*wR*(*F* ^2^) = 0.139
*S* = 1.05
7670 reflections
315 parameters
H atoms treated by a mixture of independent and constrained refinement
Δρ_max_ = 0.68 e Å^−3^
Δρ_min_ = −0.25 e Å^−3^

### 

Data collection: *APEX2* (Bruker, 2005 ▶); cell refinement: *SAINT* (Bruker, 2005 ▶); data reduction: *SAINT*; program(s) used to solve structure: *SHELXTL* (Sheldrick, 2008 ▶); program(s) used to refine structure: *SHELXTL*; molecular graphics: *SHELXTL*; software used to prepare material for publication: *SHELXTL* and *PLATON* (Spek, 2009 ▶).

## Supplementary Material

Crystal structure: contains datablocks global, I. DOI: 10.1107/S1600536809021412/bi2372sup1.cif

Structure factors: contains datablocks I. DOI: 10.1107/S1600536809021412/bi2372Isup2.hkl

Additional supplementary materials:  crystallographic information; 3D view; checkCIF report

## Figures and Tables

**Table 1:** Hydrogen-bond geometry (Å, °)

*D*—H⋯*A*	*D*—H	H⋯*A*	*D*⋯*A*	*D*—H⋯*A*
O2*A*—H1*OA*⋯O1*B*^i^	0.81	1.76	2.5608 (13)	166
O2*B*—H1*OB*⋯O1*A*^ii^	0.83	1.89	2.7118 (12)	170
N2*A*—H1*NA*⋯O4*A*	0.89 (2)	1.93 (2)	2.6332 (13)	135.3 (17)
N2*B*—H1*NB*⋯O4*B*	0.80 (2)	2.05 (2)	2.6432 (13)	130.7 (18)
C8*B*—H8*BB*⋯O4*A*^iii^	0.97	2.54	3.4613 (16)	158
C8*B*—H8*BA*⋯*Cg*1^iv^	0.97	2.83	3.4744 (13)	124

Symmetry codes: (i) 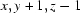; (ii) 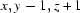; (iii) 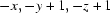; (iv) 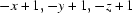. *Cg*1 is the centroid of the C1*B*–C6*B* benzene ring.

**Fig. 1.**
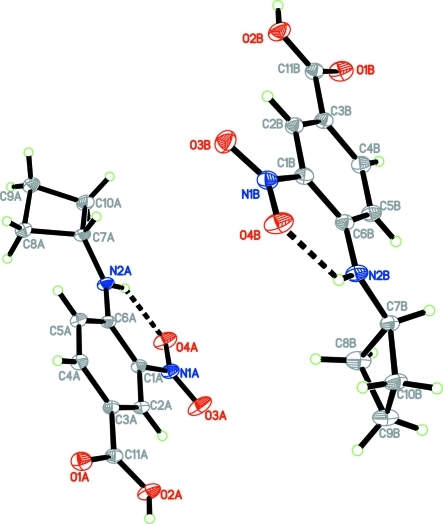
Molecular structure of the title compound, showing 50% probability displacement ellipsoids for non-H atoms. The dashed lines indicate hydrogen bonds.

**Fig. 2.**
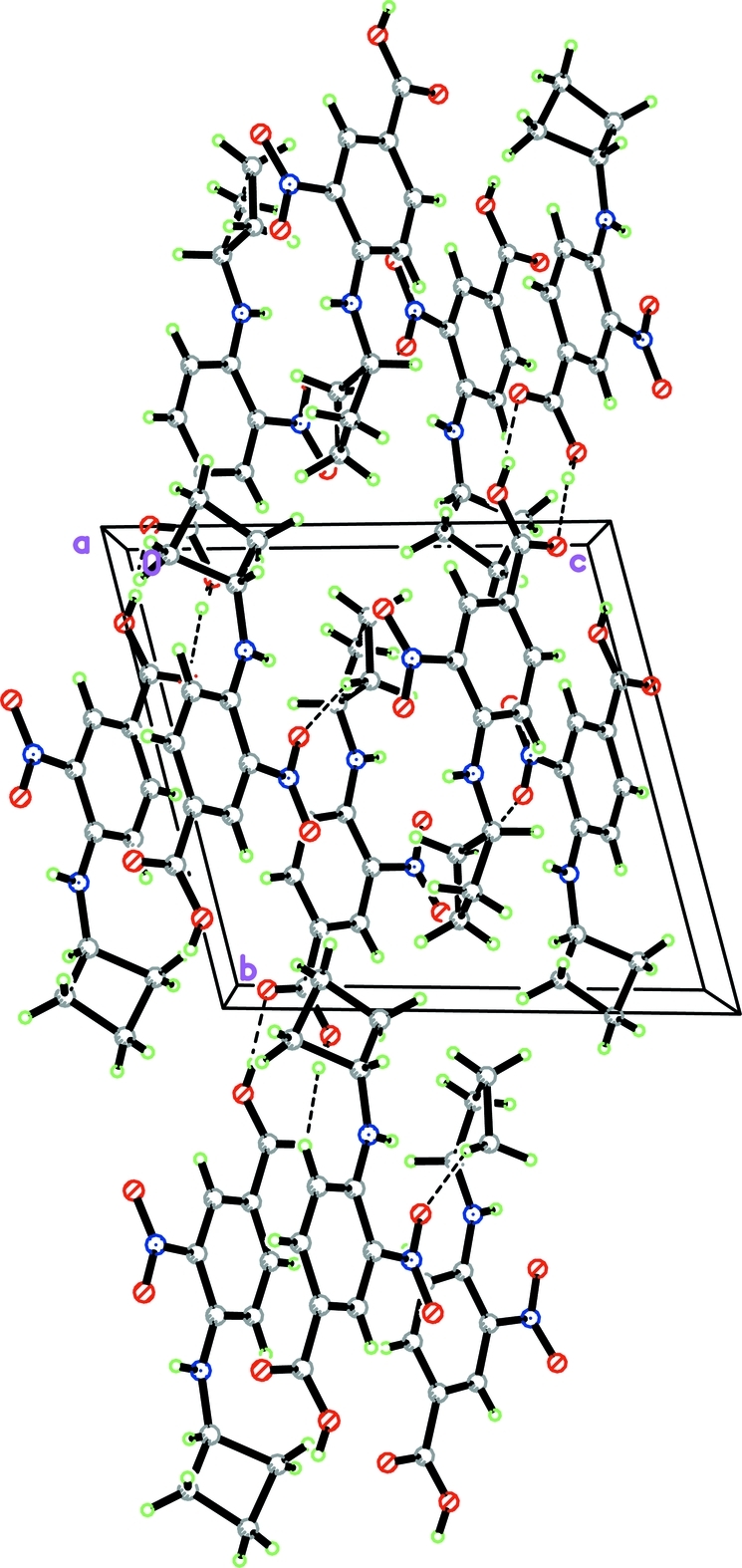
Packing diagram viewed along the a axis. Intermolecular interactions are shown as dashed lines.

## supplementary crystallographic information

### Comment

Multi functionalized benzimidazole remains as an attractive scaffold to display essential binding moieties against many validated biological targets (Wright, 1951; Singh *et al.*, 2009). This heterocycle is commonly accessed *via* nitro benzoic acid derivatives, which form a part of our current synthetic chemistry work (Narendra Babu *et al.*, 2009*a*&*b*; Ishida *et al.*, 2006). Recently, we have successfully synthesized the title compound, whose crystal structure is described here.

The asymmetric unit contains two crystallographically independent molecules (Fig. 1), *A* and *B* with similar geometries. An intramolecular N–H···O hydrogen bond is formed in both independent molecules. The dihedral angle formed by the C1A–C6A benzene ring and C7A–C10A cyclobutane is 38.29 (7)° and that between the C1B–C6B benzene ring and C7B–C10B cyclobutane is 57.04 (8)°. The nitro group in each molecule is slightly twisted away from the attached benzene ring as indicated by the torsion angle O3—N1—C1—C2, being 7.97 (15)° and 7.80 (14)° in molecules *A* and *B*, respectively.

The crystal packing (Fig. 2) is consolidated by intermolecular O—H···O and C—H···O hydrogen bonds. Molecules are linked by O—H···O hydrogen bonds between their carboxylate groups to form dimers. The crystal structure is further stabilized by C—H···π (Table 1) interactions involving the C1B–C6B benzene ring (centroid *Cg*1) and short O4B···O4B contacts (symmetry code: 2 - *x*, 1 - *y*, 1 - *z*) with distance = 2.8957 (12) Å which is shorter than the sum of van der Waals radii of the O atoms.

### Experimental

The title compound was obtained by refluxing ethyl 4-(cyclobutylamino)-3-nitro-benzoate (0.2 g, 0.0007 mol) and KOH (0.08 g, 0.0015 mol) in aqueous ethanol (5 ml) for 3 h. After completion of the reaction, ethanol was distilled off and the reaction mixture was diluted with water (5 ml). The aqueous layer was washed with dichloromethane (2 × 5 ml) and acidified with concentrated hydrochloric acid to afford a yellow solid. Yellow crystals suitable for X-ray analysis were obtained after recrystallization of the crude product with hot ethyl acetate.

### Refinement

H atoms bound to N and O atoms were located from difference Fourier maps. Atoms H1NA and H1NB were refined freely, while atoms H1OA and H1OB were refined as riding on the parent O atom with *U*_iso_(H) = 1.5 *U*_eq_(O). The remaining H atoms were positioned geometrically and refined using a riding model, with C—H = 0.93–0.98 Å and *U*_iso_(H) = 1.2 or 1.5 *U*_eq_(C).

### Crystal data

**Table:**

C_11_H_12_N_2_O_4_	*Z* = 4
*M**_r_* = 236.23	*F*(000) = 496
Triclinic, *P*1	*D*_x_ = 1.467 Mg m^−^^3^
Hall symbol: -P 1	Mo *K*α radiation, λ = 0.71073 Å
*a* = 9.8555 (2) Å	Cell parameters from 9970 reflections
*b* = 10.5308 (2) Å	θ = 2.1–34.1°
*c* = 10.9110 (2) Å	µ = 0.11 mm^−^^1^
α = 74.860 (1)°	*T* = 110 K
β = 78.265 (1)°	Block, yellow
γ = 84.826 (1)°	0.37 × 0.23 × 0.21 mm
*V* = 1069.44 (4) Å^3^	

### Data collection

**Table:**

Bruker SMART APEXII CCD diffractometer	7670 independent reflections
Radiation source: fine-focus sealed tube	6094 reflections with *I* > 2σ(*I*)
graphite	*R*_int_ = 0.027
φ and ω scans	θ_max_ = 32.5°, θ_min_ = 2.0°
Absorption correction: multi-scan (*SADABS*; Bruker, 2005)	*h* = −13→14
*T*_min_ = 0.960, *T*_max_ = 0.976	*k* = −15→15
29344 measured reflections	*l* = −16→16

### Refinement

**Table:**

Refinement on *F*^2^	Primary atom site location: structure-invariant direct methods
Least-squares matrix: full	Secondary atom site location: difference Fourier map
*R*[*F*^2^ > 2σ(*F*^2^)] = 0.048	Hydrogen site location: inferred from neighbouring sites
*wR*(*F*^2^) = 0.139	H atoms treated by a mixture of independent and constrained refinement
*S* = 1.05	*w* = 1/[σ^2^(*F*_o_^2^) + (0.079*P*)^2^ + 0.2648*P*] where *P* = (*F*_o_^2^ + 2*F*_c_^2^)/3
7670 reflections	(Δ/σ)_max_ < 0.001
315 parameters	Δρ_max_ = 0.68 e Å^−^^3^
0 restraints	Δρ_min_ = −0.25 e Å^−^^3^

### Special details

**Table:**

Experimental. The crystal was placed in the cold stream of an Oxford Cyrosystems Cobra open-flow nitrogen cryostat operating at 110.0 (1) K. Cosier, J. & Glazer, A. M. (1986). *J. Appl. Cryst.*19, 105–107.
Geometry. All e.s.d.'s (except the e.s.d. in the dihedral angle between two l.s. planes) are estimated using the full covariance matrix. The cell e.s.d.'s are taken into account individually in the estimation of e.s.d.'s in distances, angles and torsion angles; correlations between e.s.d.'s in cell parameters are only used when they are defined by crystal symmetry. An approximate (isotropic) treatment of cell e.s.d.'s is used for estimating e.s.d.'s involving l.s. planes.
Refinement. Refinement of *F*^2^ against ALL reflections. The weighted *R*-factor *wR* and goodness of fit *S* are based on *F*^2^, conventional *R*-factors *R* are based on *F*, with *F* set to zero for negative *F*^2^. The threshold expression of *F*^2^ > σ(*F*^2^) is used only for calculating *R*-factors(gt) *etc*. and is not relevant to the choice of reflections for refinement. *R*-factors based on *F*^2^ are statistically about twice as large as those based on *F*, and *R*- factors based on ALL data will be even larger.

### Fractional atomic coordinates and isotropic or equivalent isotropic displacement parameters (Å^2^)

**Table:**

	*x*	*y*	*z*	*U*_iso_*/*U*_eq_	
O1A	0.41436 (8)	0.68803 (8)	−0.08160 (8)	0.02153 (17)	
O2A	0.24499 (9)	0.80673 (8)	0.01400 (9)	0.02327 (17)	
H1OA	0.2969	0.8671	−0.0208	0.035*	
O3A	−0.16874 (10)	0.63616 (9)	0.25760 (10)	0.0315 (2)	
O4A	−0.22880 (9)	0.43744 (8)	0.28295 (10)	0.02693 (19)	
N1A	−0.14187 (9)	0.52458 (9)	0.24261 (9)	0.01836 (18)	
N2A	−0.04041 (10)	0.25875 (8)	0.22307 (9)	0.01699 (17)	
C1A	−0.00540 (10)	0.49386 (10)	0.17520 (10)	0.01442 (17)	
C2A	0.07959 (10)	0.59982 (10)	0.11780 (10)	0.01488 (18)	
H2AA	0.0469	0.6840	0.1240	0.018*	
C3A	0.21236 (10)	0.58164 (10)	0.05150 (10)	0.01456 (17)	
C4A	0.26136 (10)	0.45284 (10)	0.04592 (10)	0.01638 (18)	
H4AA	0.3507	0.4394	0.0023	0.020*	
C5A	0.17907 (11)	0.34694 (10)	0.10395 (10)	0.01650 (18)	
H5AA	0.2147	0.2629	0.1001	0.020*	
C6A	0.04005 (10)	0.36263 (9)	0.17013 (9)	0.01448 (17)	
C7A	0.00443 (12)	0.12158 (10)	0.24008 (10)	0.01755 (19)	
H7AA	0.0805	0.1007	0.2885	0.021*	
C8A	0.03497 (12)	0.06059 (10)	0.12218 (11)	0.0202 (2)	
H8AA	−0.0060	0.1105	0.0496	0.024*	
H8AB	0.1325	0.0396	0.0947	0.024*	
C9A	−0.04894 (13)	−0.05907 (11)	0.20696 (11)	0.0229 (2)	
H9AA	−0.1156	−0.0859	0.1652	0.027*	
H9AB	0.0080	−0.1337	0.2436	0.027*	
C10A	−0.11338 (13)	0.02595 (11)	0.30201 (11)	0.0235 (2)	
H10A	−0.2046	0.0642	0.2902	0.028*	
H10B	−0.1131	−0.0179	0.3920	0.028*	
C11A	0.29658 (10)	0.69811 (10)	−0.00959 (10)	0.01597 (18)	
O1B	0.37447 (9)	0.02069 (9)	0.91518 (9)	0.02540 (18)	
O2B	0.55207 (10)	−0.08564 (8)	0.81460 (9)	0.02504 (18)	
H1OB	0.5107	−0.1537	0.8547	0.038*	
O3B	0.91441 (9)	0.17243 (8)	0.51738 (8)	0.02423 (18)	
O4B	0.93948 (9)	0.37738 (8)	0.50719 (9)	0.02599 (19)	
N1B	0.87317 (9)	0.27498 (9)	0.54966 (9)	0.01787 (17)	
N2B	0.73342 (10)	0.51524 (9)	0.61428 (9)	0.01799 (17)	
C1B	0.74352 (10)	0.27632 (10)	0.63934 (10)	0.01530 (18)	
C2B	0.68286 (11)	0.15493 (10)	0.69549 (10)	0.01604 (18)	
H2BA	0.7277	0.0790	0.6762	0.019*	
C3B	0.55681 (11)	0.14668 (10)	0.77957 (10)	0.01613 (18)	
C4B	0.48894 (11)	0.26379 (10)	0.80444 (10)	0.01760 (19)	
H4BA	0.4031	0.2595	0.8595	0.021*	
C5B	0.54711 (11)	0.38380 (10)	0.74909 (10)	0.01740 (19)	
H5BA	0.4991	0.4591	0.7669	0.021*	
C6B	0.67903 (10)	0.39638 (10)	0.66502 (10)	0.01536 (18)	
C7B	0.65788 (11)	0.63794 (10)	0.62404 (10)	0.01690 (18)	
H7BA	0.6232	0.6385	0.7147	0.020*	
C8B	0.54323 (12)	0.68736 (12)	0.54265 (12)	0.0239 (2)	
H8BA	0.5534	0.6522	0.4674	0.029*	
H8BB	0.4497	0.6780	0.5926	0.029*	
C9B	0.59819 (13)	0.82720 (11)	0.51283 (13)	0.0249 (2)	
H9BA	0.6070	0.8749	0.4228	0.030*	
H9BB	0.5483	0.8803	0.5693	0.030*	
C10B	0.73574 (12)	0.76364 (10)	0.55077 (11)	0.0208 (2)	
H10C	0.8066	0.7525	0.4780	0.025*	
H10D	0.7719	0.8058	0.6059	0.025*	
C11B	0.49108 (11)	0.01977 (10)	0.83986 (10)	0.01689 (19)	
H1NA	−0.124 (2)	0.2777 (18)	0.2638 (18)	0.038 (5)*	
H1NB	0.805 (2)	0.5208 (18)	0.5636 (18)	0.035 (5)*	

### Atomic displacement parameters (Å^2^)

**Table:**

	*U*^11^	*U*^22^	*U*^33^	*U*^12^	*U*^13^	*U*^23^
O1A	0.0161 (3)	0.0208 (4)	0.0240 (4)	−0.0040 (3)	0.0020 (3)	−0.0025 (3)
O2A	0.0194 (4)	0.0128 (3)	0.0356 (5)	−0.0029 (3)	−0.0012 (3)	−0.0049 (3)
O3A	0.0237 (4)	0.0165 (4)	0.0500 (6)	−0.0019 (3)	0.0087 (4)	−0.0125 (4)
O4A	0.0160 (4)	0.0180 (4)	0.0415 (5)	−0.0058 (3)	0.0052 (3)	−0.0044 (3)
N1A	0.0148 (4)	0.0143 (4)	0.0234 (4)	−0.0015 (3)	0.0000 (3)	−0.0027 (3)
N2A	0.0180 (4)	0.0103 (4)	0.0205 (4)	−0.0027 (3)	−0.0002 (3)	−0.0020 (3)
C1A	0.0128 (4)	0.0122 (4)	0.0169 (4)	−0.0012 (3)	−0.0014 (3)	−0.0021 (3)
C2A	0.0150 (4)	0.0114 (4)	0.0173 (4)	−0.0014 (3)	−0.0031 (3)	−0.0015 (3)
C3A	0.0140 (4)	0.0120 (4)	0.0167 (4)	−0.0026 (3)	−0.0029 (3)	−0.0011 (3)
C4A	0.0141 (4)	0.0151 (4)	0.0194 (4)	−0.0003 (3)	−0.0025 (3)	−0.0039 (3)
C5A	0.0166 (4)	0.0123 (4)	0.0200 (4)	0.0002 (3)	−0.0033 (3)	−0.0032 (3)
C6A	0.0164 (4)	0.0116 (4)	0.0149 (4)	−0.0020 (3)	−0.0036 (3)	−0.0014 (3)
C7A	0.0228 (5)	0.0102 (4)	0.0193 (4)	−0.0021 (3)	−0.0047 (4)	−0.0019 (3)
C8A	0.0239 (5)	0.0149 (4)	0.0215 (5)	−0.0003 (4)	−0.0036 (4)	−0.0043 (4)
C9A	0.0327 (6)	0.0137 (4)	0.0242 (5)	−0.0039 (4)	−0.0082 (4)	−0.0049 (4)
C10A	0.0311 (6)	0.0140 (4)	0.0233 (5)	−0.0083 (4)	0.0007 (4)	−0.0030 (4)
C11A	0.0149 (4)	0.0140 (4)	0.0183 (4)	−0.0026 (3)	−0.0036 (3)	−0.0018 (3)
O1B	0.0196 (4)	0.0211 (4)	0.0306 (4)	−0.0075 (3)	0.0025 (3)	−0.0011 (3)
O2B	0.0304 (4)	0.0145 (4)	0.0284 (4)	−0.0049 (3)	−0.0012 (3)	−0.0040 (3)
O3B	0.0265 (4)	0.0204 (4)	0.0240 (4)	−0.0019 (3)	0.0033 (3)	−0.0082 (3)
O4B	0.0185 (4)	0.0176 (4)	0.0358 (5)	−0.0053 (3)	0.0044 (3)	−0.0016 (3)
N1B	0.0166 (4)	0.0166 (4)	0.0183 (4)	−0.0021 (3)	−0.0013 (3)	−0.0017 (3)
N2B	0.0160 (4)	0.0133 (4)	0.0221 (4)	−0.0024 (3)	−0.0012 (3)	−0.0011 (3)
C1B	0.0137 (4)	0.0145 (4)	0.0164 (4)	−0.0030 (3)	−0.0017 (3)	−0.0016 (3)
C2B	0.0170 (4)	0.0133 (4)	0.0174 (4)	−0.0025 (3)	−0.0031 (3)	−0.0025 (3)
C3B	0.0164 (4)	0.0134 (4)	0.0177 (4)	−0.0040 (3)	−0.0029 (3)	−0.0014 (3)
C4B	0.0154 (4)	0.0164 (4)	0.0191 (4)	−0.0025 (3)	−0.0011 (3)	−0.0022 (4)
C5B	0.0157 (4)	0.0146 (4)	0.0200 (5)	−0.0016 (3)	−0.0015 (3)	−0.0022 (3)
C6B	0.0153 (4)	0.0134 (4)	0.0169 (4)	−0.0020 (3)	−0.0043 (3)	−0.0015 (3)
C7B	0.0185 (4)	0.0129 (4)	0.0186 (4)	−0.0014 (3)	−0.0043 (4)	−0.0017 (3)
C8B	0.0188 (5)	0.0209 (5)	0.0304 (6)	−0.0031 (4)	−0.0089 (4)	0.0008 (4)
C9B	0.0241 (5)	0.0166 (5)	0.0308 (6)	−0.0003 (4)	−0.0080 (4)	0.0018 (4)
C10B	0.0221 (5)	0.0131 (4)	0.0270 (5)	−0.0038 (4)	−0.0080 (4)	−0.0011 (4)
C11B	0.0168 (4)	0.0145 (4)	0.0186 (4)	−0.0039 (3)	−0.0028 (3)	−0.0022 (3)

### Geometric parameters (Å, °)

**Table:**

O1A—C11A	1.2752 (13)	O1B—C11B	1.2705 (13)
O2A—C11A	1.2763 (12)	O2B—C11B	1.2812 (13)
O2A—H1OA	0.815	O2B—H1OB	0.829
O3A—N1A	1.2264 (12)	O3B—N1B	1.2316 (12)
O4A—N1A	1.2439 (12)	O4B—N1B	1.2440 (12)
N1A—C1A	1.4484 (13)	N1B—C1B	1.4442 (13)
N2A—C6A	1.3413 (13)	N2B—C6B	1.3412 (13)
N2A—C7A	1.4472 (13)	N2B—C7B	1.4497 (13)
N2A—H1NA	0.888 (19)	N2B—H1NB	0.802 (19)
C1A—C2A	1.3889 (14)	C1B—C2B	1.3952 (14)
C1A—C6A	1.4254 (13)	C1B—C6B	1.4313 (14)
C2A—C3A	1.3836 (14)	C2B—C3B	1.3808 (14)
C2A—H2AA	0.930	C2B—H2BA	0.930
C3A—C4A	1.4105 (13)	C3B—C4B	1.4122 (14)
C3A—C11A	1.4728 (14)	C3B—C11B	1.4704 (14)
C4A—C5A	1.3740 (14)	C4B—C5B	1.3718 (14)
C4A—H4AA	0.930	C4B—H4BA	0.930
C5A—C6A	1.4300 (14)	C5B—C6B	1.4254 (14)
C5A—H5AA	0.930	C5B—H5BA	0.930
C7A—C10A	1.5347 (15)	C7B—C10B	1.5365 (15)
C7A—C8A	1.5483 (15)	C7B—C8B	1.5482 (15)
C7A—H7AA	0.980	C7B—H7BA	0.980
C8A—C9A	1.5477 (16)	C8B—C9B	1.5458 (17)
C8A—H8AA	0.970	C8B—H8BA	0.970
C8A—H8AB	0.970	C8B—H8BB	0.970
C9A—C10A	1.5476 (16)	C9B—C10B	1.5418 (16)
C9A—H9AA	0.970	C9B—H9BA	0.970
C9A—H9AB	0.970	C9B—H9BB	0.970
C10A—H10A	0.970	C10B—H10C	0.970
C10A—H10B	0.970	C10B—H10D	0.970
			
C11A—O2A—H1OA	111.9	C11B—O2B—H1OB	114.1
O3A—N1A—O4A	121.83 (9)	O3B—N1B—O4B	122.07 (9)
O3A—N1A—C1A	119.01 (9)	O3B—N1B—C1B	118.88 (9)
O4A—N1A—C1A	119.16 (9)	O4B—N1B—C1B	119.05 (9)
C6A—N2A—C7A	126.24 (9)	C6B—N2B—C7B	123.78 (9)
C6A—N2A—H1NA	114.6 (12)	C6B—N2B—H1NB	118.5 (13)
C7A—N2A—H1NA	118.1 (12)	C7B—N2B—H1NB	116.5 (13)
C2A—C1A—C6A	121.97 (9)	C2B—C1B—C6B	122.19 (9)
C2A—C1A—N1A	115.91 (9)	C2B—C1B—N1B	116.19 (9)
C6A—C1A—N1A	122.12 (9)	C6B—C1B—N1B	121.58 (9)
C3A—C2A—C1A	120.77 (9)	C3B—C2B—C1B	120.48 (9)
C3A—C2A—H2AA	119.6	C3B—C2B—H2BA	119.8
C1A—C2A—H2AA	119.6	C1B—C2B—H2BA	119.8
C2A—C3A—C4A	118.77 (9)	C2B—C3B—C4B	118.68 (9)
C2A—C3A—C11A	118.30 (9)	C2B—C3B—C11B	121.37 (9)
C4A—C3A—C11A	122.93 (9)	C4B—C3B—C11B	119.92 (9)
C5A—C4A—C3A	121.02 (9)	C5B—C4B—C3B	121.34 (10)
C5A—C4A—H4AA	119.5	C5B—C4B—H4BA	119.3
C3A—C4A—H4AA	119.5	C3B—C4B—H4BA	119.3
C4A—C5A—C6A	121.66 (9)	C4B—C5B—C6B	121.84 (9)
C4A—C5A—H5AA	119.2	C4B—C5B—H5BA	119.1
C6A—C5A—H5AA	119.2	C6B—C5B—H5BA	119.1
N2A—C6A—C1A	123.24 (9)	N2B—C6B—C5B	120.09 (9)
N2A—C6A—C5A	120.98 (9)	N2B—C6B—C1B	124.48 (9)
C1A—C6A—C5A	115.78 (9)	C5B—C6B—C1B	115.42 (9)
N2A—C7A—C10A	113.66 (9)	N2B—C7B—C10B	115.50 (9)
N2A—C7A—C8A	119.61 (9)	N2B—C7B—C8B	118.69 (9)
C10A—C7A—C8A	89.08 (8)	C10B—C7B—C8B	88.50 (8)
N2A—C7A—H7AA	110.9	N2B—C7B—H7BA	110.8
C10A—C7A—H7AA	110.9	C10B—C7B—H7BA	110.8
C8A—C7A—H7AA	110.9	C8B—C7B—H7BA	110.8
C9A—C8A—C7A	88.22 (8)	C9B—C8B—C7B	87.84 (8)
C9A—C8A—H8AA	113.9	C9B—C8B—H8BA	114.0
C7A—C8A—H8AA	113.9	C7B—C8B—H8BA	114.0
C9A—C8A—H8AB	113.9	C9B—C8B—H8BB	114.0
C7A—C8A—H8AB	113.9	C7B—C8B—H8BB	114.0
H8AA—C8A—H8AB	111.2	H8BA—C8B—H8BB	111.2
C10A—C9A—C8A	88.63 (8)	C10B—C9B—C8B	88.40 (8)
C10A—C9A—H9AA	113.9	C10B—C9B—H9BA	113.9
C8A—C9A—H9AA	113.9	C8B—C9B—H9BA	113.9
C10A—C9A—H9AB	113.9	C10B—C9B—H9BB	113.9
C8A—C9A—H9AB	113.9	C8B—C9B—H9BB	113.9
H9AA—C9A—H9AB	111.1	H9BA—C9B—H9BB	111.1
C7A—C10A—C9A	88.71 (9)	C7B—C10B—C9B	88.40 (8)
C7A—C10A—H10A	113.9	C7B—C10B—H10C	113.9
C9A—C10A—H10A	113.9	C9B—C10B—H10C	113.9
C7A—C10A—H10B	113.9	C7B—C10B—H10D	113.9
C9A—C10A—H10B	113.9	C9B—C10B—H10D	113.9
H10A—C10A—H10B	111.1	H10C—C10B—H10D	111.1
O1A—C11A—O2A	123.11 (10)	O1B—C11B—O2B	122.99 (10)
O1A—C11A—C3A	120.54 (9)	O1B—C11B—C3B	117.51 (9)
O2A—C11A—C3A	116.35 (9)	O2B—C11B—C3B	119.49 (9)
			
O3A—N1A—C1A—C2A	−7.97 (15)	O3B—N1B—C1B—C2B	7.80 (14)
O4A—N1A—C1A—C2A	171.59 (10)	O4B—N1B—C1B—C2B	−172.39 (10)
O3A—N1A—C1A—C6A	170.85 (10)	O3B—N1B—C1B—C6B	−169.99 (10)
O4A—N1A—C1A—C6A	−9.59 (15)	O4B—N1B—C1B—C6B	9.82 (15)
C6A—C1A—C2A—C3A	0.82 (16)	C6B—C1B—C2B—C3B	−0.32 (16)
N1A—C1A—C2A—C3A	179.65 (9)	N1B—C1B—C2B—C3B	−178.10 (9)
C1A—C2A—C3A—C4A	−1.57 (15)	C1B—C2B—C3B—C4B	1.83 (15)
C1A—C2A—C3A—C11A	178.78 (9)	C1B—C2B—C3B—C11B	−179.98 (10)
C2A—C3A—C4A—C5A	0.59 (15)	C2B—C3B—C4B—C5B	−1.38 (16)
C11A—C3A—C4A—C5A	−179.77 (10)	C11B—C3B—C4B—C5B	−179.61 (10)
C3A—C4A—C5A—C6A	1.17 (16)	C3B—C4B—C5B—C6B	−0.62 (16)
C7A—N2A—C6A—C1A	−169.40 (10)	C7B—N2B—C6B—C5B	−9.26 (16)
C7A—N2A—C6A—C5A	10.71 (16)	C7B—N2B—C6B—C1B	169.59 (10)
C2A—C1A—C6A—N2A	−179.01 (10)	C4B—C5B—C6B—N2B	−179.01 (10)
N1A—C1A—C6A—N2A	2.23 (16)	C4B—C5B—C6B—C1B	2.04 (15)
C2A—C1A—C6A—C5A	0.88 (15)	C2B—C1B—C6B—N2B	179.51 (10)
N1A—C1A—C6A—C5A	−177.88 (9)	N1B—C1B—C6B—N2B	−2.83 (16)
C4A—C5A—C6A—N2A	178.03 (10)	C2B—C1B—C6B—C5B	−1.59 (15)
C4A—C5A—C6A—C1A	−1.86 (15)	N1B—C1B—C6B—C5B	176.07 (9)
C6A—N2A—C7A—C10A	−179.08 (10)	C6B—N2B—C7B—C10B	−175.71 (10)
C6A—N2A—C7A—C8A	−75.85 (14)	C6B—N2B—C7B—C8B	−72.54 (14)
N2A—C7A—C8A—C9A	−134.34 (10)	N2B—C7B—C8B—C9B	−138.08 (10)
C10A—C7A—C8A—C9A	−17.43 (9)	C10B—C7B—C8B—C9B	−19.62 (9)
C7A—C8A—C9A—C10A	17.28 (9)	C7B—C8B—C9B—C10B	19.55 (9)
N2A—C7A—C10A—C9A	139.60 (9)	N2B—C7B—C10B—C9B	140.96 (10)
C8A—C7A—C10A—C9A	17.42 (8)	C8B—C7B—C10B—C9B	19.66 (9)
C8A—C9A—C10A—C7A	−17.43 (9)	C8B—C9B—C10B—C7B	−19.70 (9)
C2A—C3A—C11A—O1A	−173.48 (10)	C2B—C3B—C11B—O1B	−179.36 (10)
C4A—C3A—C11A—O1A	6.87 (16)	C4B—C3B—C11B—O1B	−1.19 (15)
C2A—C3A—C11A—O2A	6.58 (14)	C2B—C3B—C11B—O2B	0.37 (16)
C4A—C3A—C11A—O2A	−173.06 (10)	C4B—C3B—C11B—O2B	178.54 (10)

### Hydrogen-bond geometry (Å, °)

**Table:**

*D*—H···*A*	*D*—H	H···*A*	*D*···*A*	*D*—H···*A*
O2A—H1OA···O1B^i^	0.81	1.76	2.5608 (13)	166
O2B—H1OB···O1A^ii^	0.83	1.89	2.7118 (12)	170
N2A—H1NA···O4A	0.89 (2)	1.93 (2)	2.6332 (13)	135.3 (17)
N2B—H1NB···O4B	0.80 (2)	2.05 (2)	2.6432 (13)	130.7 (18)
C8B—H8BB···O4A^iii^	0.97	2.54	3.4613 (16)	158
C8B—H8BA···Cg1^iv^	0.97	2.83	3.4744 (13)	124

Symmetry codes: (i) *x*, *y*+1, *z*−1; (ii) *x*, *y*−1, *z*+1; (iii) −*x*, −*y*+1, −*z*+1; (iv) −*x*+1, −*y*+1, −*z*+1.
